# Compensatory Upregulation of Anti-Beta-Adrenergic Receptor Antibody Levels Might Prevent Heart Failure Presentation in Pediatric Myocarditis

**DOI:** 10.3389/fped.2022.881208

**Published:** 2022-04-28

**Authors:** Franziska Seidel, Carmen Scheibenbogen, Harald Heidecke, Bernd Opgen-Rhein, Thomas Pickardt, Karin Klingel, Felix Berger, Daniel Messroghli, Stephan Schubert

**Affiliations:** ^1^German Heart Center Berlin, Department of Congenital Heart Disease and Pediatric Cardiology, Berlin, Germany; ^2^Charité – Universitätsmedizin Berlin, Department of Pediatric Cardiology, Berlin, Germany; ^3^Experimental and Clinical Research Center, a Cooperation between the Max-Delbrück-Center for Molecular Medicine in the Helmholtz Association and the Charité – Universitätsmedizin Berlin, Berlin, Germany; ^4^Charité – Universitätsmedizin Berlin, Institute for Imaging Science and Computational Modelling in Cardiovascular Medicine, Berlin, Germany; ^5^DZHK (German Centre for Cardiovascular Research), Partner Site Berlin, Berlin, Germany; ^6^Charité – Universitätsmedizin Berlin, Outpatient Clinic for Immunodeficiencies, Institute for Medical Immunology, Berlin, Germany; ^7^CellTrend GmbH, Luckenwalde, Germany; ^8^Competence Network for Congenital Heart Diseases, Berlin, Germany; ^9^Department of Cardiopathology, Institute for Pathology and Neuropathology, University Hospital Tübingen, Tübingen, Germany; ^10^German Heart Center Berlin, Department of Internal Medicine – Cardiology, Berlin, Germany; ^11^Charité – Universitätsmedizin Berlin, Department of Internal Medicine and Cardiology, Berlin, Germany; ^12^Center for Congenital Heart Disease/Pediatric Cardiology, Heart and Diabetes Center NRW, University Clinic of Ruhr-University Bochum, Bad Oeynhausen, Germany

**Keywords:** anti-beta-adrenergic receptor antibodies, myocarditis, autoimmune, pediatric, endomyocardial biopsy

## Abstract

**Background:**

Myocarditis can be associated with severe heart failure and is caused by different inflammatory and autoimmune responses. The aim of this study was to describe the immunological response in children with myocarditis by analyzing anti-beta-adrenergic receptor antibodies (anti-β-AR Abs).

**Methods:**

Sera of children who were hospitalized with biopsy-proven myocarditis were prospectively collected between April 2017 and March 2019. Anti-β1-AR Ab, anti-β2-AR Ab, and anti-β3-AR Ab were quantified by a CE-certified ELISA kit. According to normal values for immunoglobulin G (IgG), three age groups, <1, 1–5, and >5–17 years, were defined. Children without inflammatory cardiac pathology and no heart failure signs were served as a control group.

**Results:**

We compared 22 patients with biopsy-proven myocarditis and 28 controls. The median age (interquartile range) of the myocarditis group (MYC) was 12.1 (2.7–16.4) years, 13 men, left ventricular ejection fraction (LVEF) 51% and for control group, the median age was 5.0 (3.0–6.8) years, nine men, LVEF 64%. Myocarditis patients in the age group >5–17 years showed significantly higher anti-β3-AR Ab levels as compared to controls (*p* = 0.014). Lower anti-β2-AR Ab and anti-β3-AR Ab levels were significantly correlated with higher left ventricular diameters in myocarditis patients. The event-free survival using a combined endpoint (mechanical circulatory support [MCS], transplantation, and/or death) was significantly lower in myocarditis patients with antibody levels below the median as compared to myocarditis patients with antibody levels ≥ the median.

**Conclusion:**

Anti-β-AR Ab levels are increased in children with myocarditis and >5 years of age. These antibodies might be upregulated compensatory to prevent further cardiac deterioration. A worse event-free survival in patients with lower anti-β-AR Ab levels might be a therapeutic target for immunoglobulin substitution.

## Introduction

Myocarditis is an inflammatory disease of the myocardium that is caused by various different triggers (1). In children, it is one major cause of the development of acute or chronic heart failure (2). One of the most striking findings in pediatric patients with myocarditis is the high prevalence of heart failure with severely reduced systolic function in patients under 1 year of age (3, 4). This specific cohort often experiences adverse events, such as the need for mechanical circulatory support (MCS) or heart transplantation (HTx) and death, whereas adolescents mostly present with preserved or mild impaired left ventricular ejection fraction (LVEF) and angina pectoris (4, 5). At the same time, studies on the therapeutic use of immunoglobulins have failed to show any positive effects in adults and adolescents but showed encouraging effects in young children (6). Both findings might be related to the fact that the immunological activities, such as immunoglobulin production, only reach a mature state at the age of >5 years (7). Focusing on anti-beta-adrenergic receptor antibodies (anti-β-AR Ab), levels have been described as increased in patients with myocarditis and dilated cardiomyopathy (DCM) and are associated with heart failure and arrhythmogenic events (8–10). In children with DCM and poor clinical outcomes, high anti-β1-AR Ab levels were described (11). However, a multicenter study in children with myocarditis could not verify this for anti-β1- and anti-β2-AR Ab (12). In adults with an ST-elevation myocardial infarct (STEMI), lowered anti-β1-AR Ab levels led to higher rates of re-infarction and cardiovascular death pointing toward a possible protective effect of these antibodies (13). Referring to these divergent results, the impact of anti-β-AR Ab remains not fully understood, especially in children.

The aim of this study was to analyze anti-β-AR Ab levels in pediatric patients with biopsy-proven myocarditis and pediatric controls to gain knowledge on their distribution in the different age groups and their impact on the outcome.

## Materials and Methods

### Patient Data and Follow-Up

Sera from patients under 18 years of age with suspected myocarditis and enrollment within the MYKKE registry between April 2017 and March 2019 were collected at the Pediatric Cardiology Departments of the German Heart Center Berlin and the Charité - Universitätsmedizin Berlin, Berlin, Germany. The study was approved by the institutional ethics committee (Charité - Universitätsmedizin Berlin, EA2/131/10, EA2/074/13). All parents or guardians of patients <18 years gave written informed consent.

Serum was collected at the time of admission, centrifuged at 20°C and 3,800 g for 10 min, and frozen at −80°C. Clinical parameters, diagnostic cardiac imaging, and endomyocardial biopsy (EMB) were assessed routinely. Initial clinical and follow-up data were entered into the online MYKKE study database (4). Patients without EMB or not proven myocarditis in EMB were excluded from further analyses.

**Figure 1:**
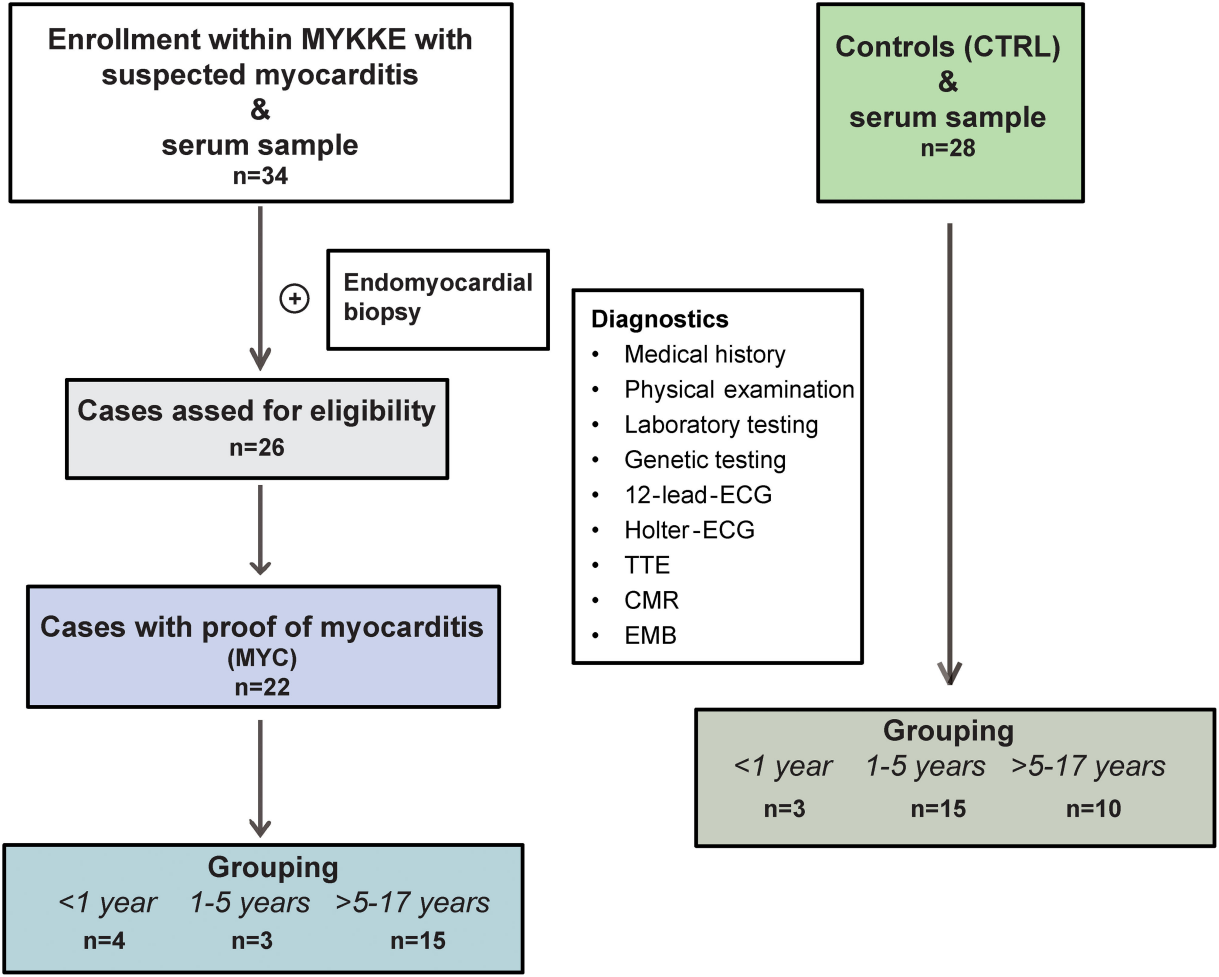
Study flow chart. Selection of enrolled patients within the MYKKE registry depends on sera withdrawal and the proof of myocarditis in the endomyocardial biopsy. Four cases diagnosed with dilated cardiomyopathy in EMB could not be included in the final analysis. Grouping was conducted according to normal values for immunoglobulin G into three age groups. CMR, cardiac magnetic resonance; ECG, electrocardiogram; EMB, endomyocardial biopsy; TTE, transthoracic echocardiography.

Patients with proof of myocarditis in EMB were called the myocarditis group (MYC). Patients under 18 years of age, administered for elective cardiac catheterization, and without inflammatory cardiac pathology served were as a control group (CTRL). See the study flow chart for further details (Figure 1).

Regarding outcomes, the occurrence of adverse events, such as MCS, HTx, and/or all-cause death, was defined as a combined endpoint.

### Detection of Anti-β-Adrenergic Antibodies

Anti-β1-AR Ab, anti-β2-AR Ab, and anti-β3-AR Ab were measured with commercially available enzyme-linked immunosorbent assays (ELISA; CellTrend GmbH, Luckenwalde, Germany) according to the instructions of the manufacturer (14). All these assays provided native receptors presented in their physiological membrane environment as immunogenic targets of immunoglobulin G (IgG) binding.

### Analysis of Endomyocardial Biopsies

All EMB specimens were analyzed histopathologically and immunohistologically and by polymerase chain reaction [(RT-)PCR] for myocardial detection of viral RNA/DNA by one specialized center for Cardiopathology (Institute for Pathology and Neuropathology, University Hospital Tübingen, Tübingen, Germany) as previously described (15).

The diagnosis of myocarditis was confirmed according to the established criteria and grouped in accordance with the WHO definition in (16, 17):

(a) Acute myocarditis: Infiltrate of ≥14 leucocytes/mm^2^ and presence of myocyte damage.

(b) Healing/chronic myocarditis: Infiltrate of ≥14 leucocytes/mm^2^ and absence of myocyte damage but the presence of fibrosis.

(c) Healed myocarditis: Multifocal fibrosis or scarring without inflammation (0–3 leucocytes/mm^2^).

### Statistical Analysis

Categorical variables were summarized by frequencies and percentages. For continuous measures, data were presented as median values with interquartile range (IQR). Pearson's chi-square test and Fisher's exact test were used to compare dichotomous variables. For comparison of independent groups, the Mann-Whitney U and Kruskal-Wallis test were applied. For correlation of antibody levels and laboratory and clinical parameters, Spearman's rho test was used. Kaplan-Meier curves and log-rank tests were employed for survival analysis. Therefore, the groups < median and ≥ median were built according to the median anti-β-AR Ab levels of the MYC. A probability value of <0.05 was considered statistically significant. Data were analyzed using IBM Corp. SPSS Version 24.0 (Armonk, NY, USA).

## Results

### Basic Characteristics

We enrolled 22 patients with biopsy-proven myocarditis and median age (IQR) of 12.1 (2.7–16.4) years, 13 were men (MYC). Twenty-eight patients served as controls with a median age of 5.0 (3.0–6.8) years, 9 were men (CTRL).

The control group was administered due to the following diagnoses: atrial septal defect (ASD; *n* = 19), persistent ductus arteriosus (PDA; *n* = 6), a combination of ASD and PDA (*n* = 1), aortic isthmus stenosis (*n* = 1), and mild pulmonary valve stenosis (*n* = 1). The MYC had significantly lower LVEF in echocardiography and higher Z-scores of the left ventricular internal diastolic diameter (LVIDd; *p* < 0.001, respectively). Detailed basic characteristics are given Table 1.

**Table 1:** Basic characteristics of the myocarditis (MYC) and control group (CTRL).

	**MYC group *n* = 22**	**CTRL group *n* = 28**	***p*-Value**
Gender, male	13 (59)	9 (32)	0.086
Age (years)	12.1 (2.7–16.4)	5.0 (3.0–6.8)	**0.042**
BSA (kg/m^2^)	1.6 (0.6–1.8)	0.8 (0.6–1.2)	0.063
**Echocardiography**
Z-score LVIDd (mm)	1.9 (0.1–5.3)	−0.8 (−1.6–0.4)	**<0.001**
LVEF (%)	51.0 (28.0–60.0)	63.5 (57.8–72.3)	**<0.001**
**Antibody levels**
Anti-β1-AR Ab (U/ml)	7.7 (3.8–24.3)	5.0 (3.7–8.2)	0.125
Anti-β2-AR Ab (U/ml)	6.0 (2.4–19.9)	3.6 (2.6–4.7)	0.077
Anti-β3-AR Ab (U/ml)	6.4 (2.9–18.8)	3.5 (3.0–6.8)	**0.035**

*Values are given as n (%) or median (interquartile range)*.

*Anti-β-AR Ab, anti-beta-adrenergic antibodies; BSA, body surface area; LVEF, left ventricular ejection fraction; LVIDd, left ventricular internal diastolic diameter. Bold values represents significant values*.

### Basic Characteristics of MYC Patients and Controls Within Age Groups

According to known values for overall IgG and its age-depending distribution in childhood, three age subgroups (<1 year, 1–5 years, and >5–17 years) were defined for both groups (MYC and CTRL, see Table 2) (7).

**Table 2:** Anti-beta-adrenergic antibody levels and echocardiographic parameters of controls (CTRL) and myocarditis (MYC) patients in different age groups.

	**Age group** ** <1 year**			**Age group 1–5 years**			**Age group** **>5–17 years**		
	**MYC group** ***n*** **=** **4**	**CTRL group** ***n*** **=** **3**	***p*** **-value**	**MYC group** ***n*** **=** **3**	**CTRL group** ***n*** **=** **15**	***p*** **-value**	**MYC group** ***n*** **=** **15**	**CTRL group** ***n*** **=** **10**	***p*** **-value**
Gender, male	0 (0)	1 (33)	0.429	3 (100)	5 (33)	0.069	12 (71)	5 (28)	0.111
Age (years)	0.4 (0.2–0.7)	0.0^*^	0.057	3.2^*^	4.0 (3.0–5.0)	0.426	15.6 (11.9–16.9)	10.5 (6.0–17.0)	**0.015**
**Echocardiography**
Z-score LVIDd (mm)	6.0 (1.5–7.9)	−1.0^*^	0.057	6.2^*^	−0.7 (−1.6-0.3)	**0.005**	0.5 (−0.4–2.4)	−1.1 (-3.1–0.9)	0.071
LVEF (%)	25.5 (14.3–36.8)	72.0^*^	0.057	22.0^*^	65.0 (57.5–73.0)	**0.004**	57.0 (50.0–61.0)	59.5 (57.0–68.5)	0.071
**Antibody levels**
Anti-β1-AR Ab (U/ml)	3.6 (1.0–13.2)	1.9^*^	0.857	8.3^*^	5.5 (4.3–10.8)	0.498	8.2 (4.5–29.8)	4.5 (3.8–6.3)	0.071
Anti-β2-AR Ab (U/ml)	1.7 (0.7–6.1)	2.7^*^	0.629	6.6^*^	3.6 (2.6–5.5)	0.130	6.2 (2.9–22.7)	3.8 (2.6–4.5)	0.080
Anti-β3-AR Ab (U/ml)	1.2 (1.0–5.8)	4.5^*^	0.400	7.2^*^	3.5 (3.0–5.3)	0.076	6.6 (3.4–19.4)	3.3 (2.4–4.5)	**0.014**
**EMB**
Acute myocarditis	1 (25)	n.a.		2 (67)	n.a.		2 (13)	n.a.	
Chronic/healing myocarditis	3 (75)	n.a.		1 (33)	n.a.		12 (80)	n.a.	
Healed myocarditis	0 (0)	n.a.		0 (0)	n.a.		1 (7)	n.a.	

*EMB, endomyocardial biopsy; MYC, patients with biopsy proven inflammatory myocardial disease; CTRL, patients without inflammatory myocardial disease. Values are given as n (%) or median (interquartile range)*.

* *Only median. Anti-β-AR Ab, anti-beta-adrenergic antibodies; BSA, body surface area; LVEF, left ventricular ejection fraction; LVIDd, left ventricular internal diastolic diameter. Bold values represents significant values. n.a.; not applicable*.

Four patients with myocarditis and three in the control group belonged to the age group < 1 year. The MYC group was presented with a severely reduced LVEF and dilated left ventricles. They were presented with signs of heart failure and a median (IQR) value of N-terminal-pro brain natriuretic peptide (NT-proBNP) of 133,389 (103,375–177,557) ng/l. Troponin Ths was increased with 1,425 ng/l. No pathologies in the blood count or C-reactive protein (CRP) increment were detected. Two had the diagnosis of healing/chronic myocarditis and one of acute myocarditis in EMB. The control group (*n* = 3) was presented with normal LVEF and had no dilated ventricles.

The age group of 1–5 years consisted of 3 MYC patients and 15 controls. Again, patients with MYC showed signs of heart failure with severely reduced LVEF and left ventricular dilatation. The median NT-proBNP was 35,000 ng/l, Troponin Ths was 85 ng/l. Leucocytes, thrombocytes, and hemoglobin were in a normal range. The CRP was slightly increased at 17.0 mg/dl. Two patients with MYC were diagnosed with acute myocarditis in EMB, one with a chronic/healing myocarditis. The controls in the age groups 1–5 years had a normal LVIDd and LVEF.

Fifteen patients with myocarditis and a median age of 15.6 (11.9–16.9) years belonged to the age group >5–17 years, 12 were men. All had biopsy-proven lymphocytic myocarditis: 12 reported chronic/healing myocarditis, 2 with acute, and 1 with healed myocarditis (see Table 2). They presented with a median Z-score of the LVIDd of 0.5 (−0.4 to 2.4) and an LVEF of 57.0 (50.0–61.0)% (18). NT-proBNP was increased at 577.0 (175.8–1885.8) ng/l and Troponin Ths with 672.0 (523.5–1427.0) ng/l. Leucocytes, thrombocytes, and hemoglobin were in a normal range, whereas CRP was increased with 37.6 (1.4–137.0) mg/l. Ten patients served as a control group with a median age of 10.5 (6.0–17.0) years, 5 were men.

The myocarditis patients in the age group >5−7 years did not differ significantly in LVEF and Z-scores of the LVIDd as compared to controls (see Table 2; *p* = 0.071, respectively).

Further, MYC patients with Anti-β3-AR Ab level ≥10 U/ml had a significantly lower Z-Score of LVIDd than MYC patients with Anti-β3-AR Ab <10 U/ml [−0.1 (−1.4 to 0.5) vs. 2.3 (0.2–3.5); *p* = 0.036]. There was no significant difference in age, body surface area, sex, LVEF, NT-proBNP, the combined endpoint, or the diagnosis in the EMB.

### Antibody Levels Within Age Groups

**Figure 2:**
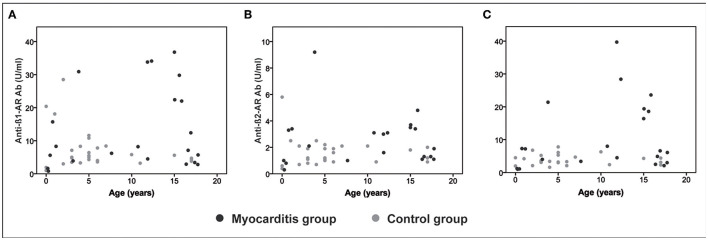
Antibodies over age. Distribution of anti-β-adrenergic antibodies (anti-β-AR Ab) over age for the myocarditis group (dark gray dots) and controls (light gray dots). **(A)** anti-β1-AR Ab. **(B)** anti-β2-AR Ab. **(C)** anti-β3-AR Ab.

The comparison of anti-β-AR Ab levels between myocarditis patients and controls within the first two age groups (<1 year and 1–5 years) did not differ significantly. Patients >5 years of age showed significantly higher anti-β3-AR Ab levels as compared to controls (*p* = 0.014; see Table 2). The distribution of the different anti-β-AR Ab levels over age are shown in Figure 2.

### Correlations of Antibody Levels and Clinical Parameters

**Figure 3:**
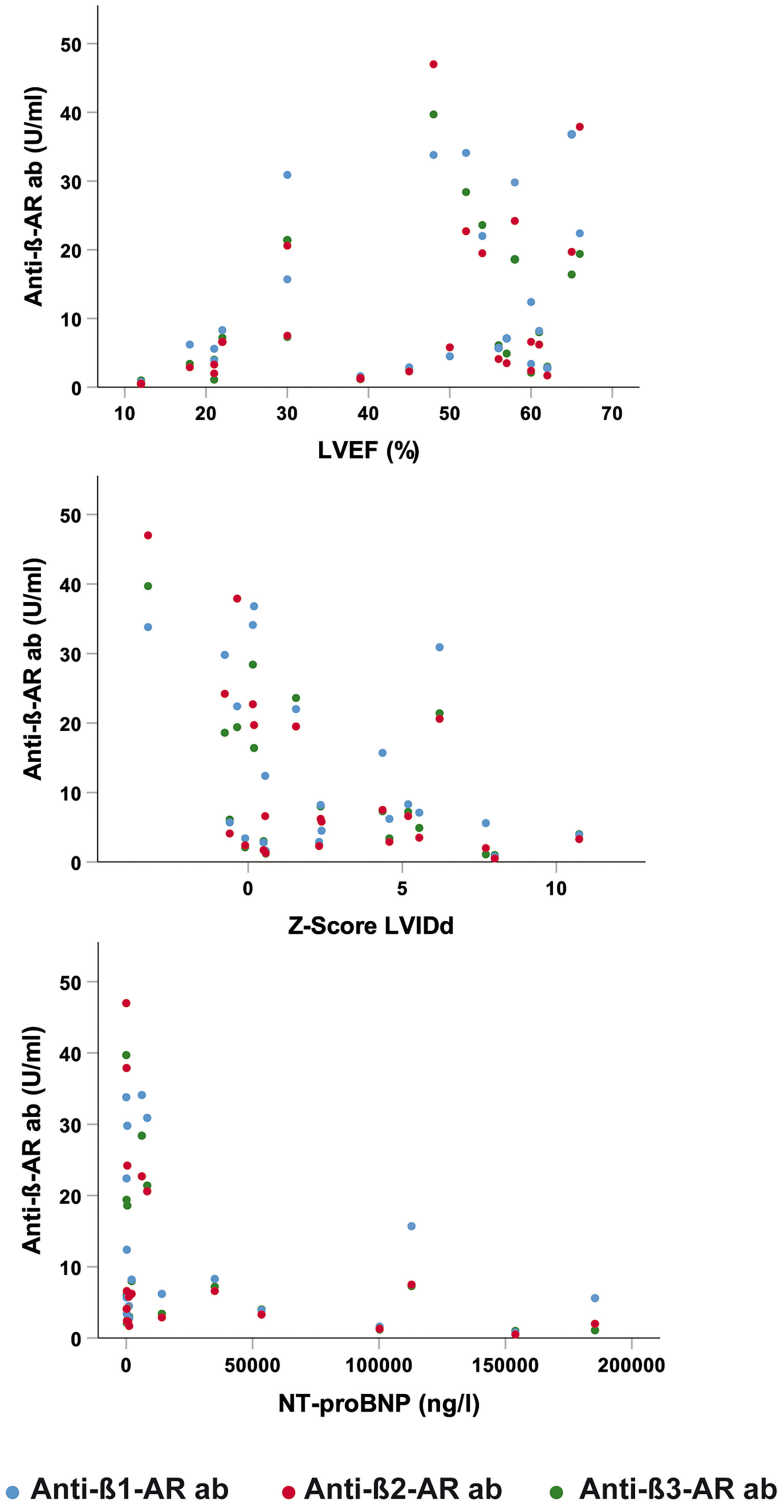
Correlation of anti-β-AR ab levels and clinical parameters. (Upper) Correlation of anti-β-AR ab levels and left ventricular ejection fraction (LVEF). (Middle) Correlation of anti-β-AR ab levels and Z-score of the left ventricular internal diastolic diameter (LVIDd). (Lower) Correlation of anti-β-AR ab levels and N-terminal-pro brain natriuretic peptide (NT-proBNP). Blue dots: anti-β1-AR ab. Red dots: anti-β2-AR ab. Green dots: anti-β3-AR ab.

Lower anti-β2-AR Ab and anti-β3-AR Ab levels were significantly correlated with increased Z-score of the LVIDd in patients with MYC across all ages (β2: *p* = 0.029; β3: *p* = 0.045, see Figure 3). An inverse correlation was detected between anti-β2-AR Ab levels and NT-proBNP (*p* = 0.034, see Figure 3). No significant correlations between anti-β-AR Ab levels and other inflammatory laboratory parameters were found in the myocarditis patients.

### Impact of Antibody Levels on the Combined Endpoint

Seven out of 22 myocarditis patients reached the combined endpoint of MCS, HTx, or death. All seven patients needed MCS, three were transplanted. No patient had died. Four patients were belonged to the age group <1 year, two to the age group 1–5 years, and one was in the age group >5–17 years.

**Figure 4:**
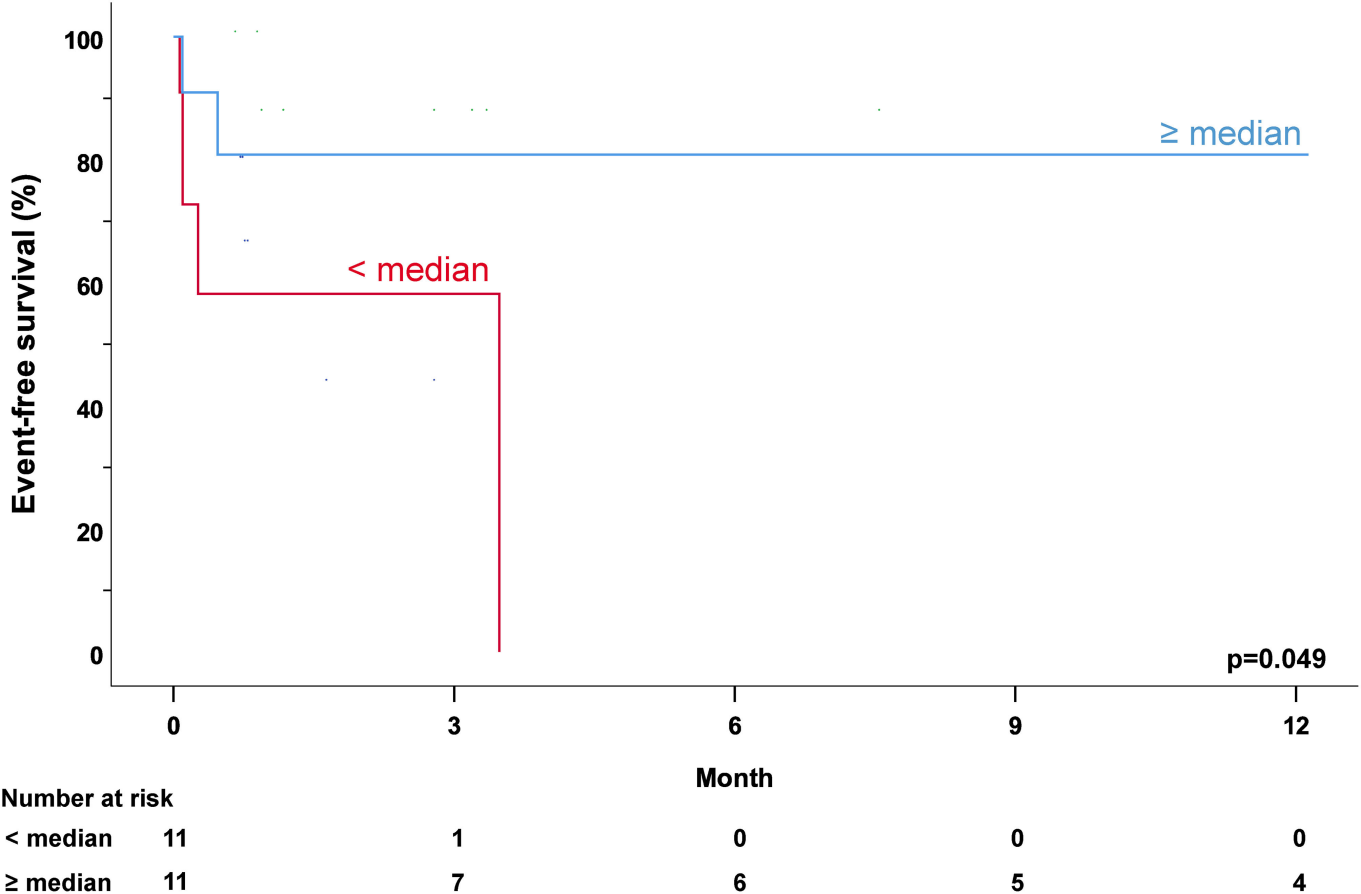
Anti-β1-adrenergic antibody levels and outcome across all age groups. Combined endpoint of mechanical circulatory support, heart transplantation, and/or death of myocarditis patients with regard to the median of anti-β1-adrenergic antibody (anti-β1-AR Ab) levels following hospital admission. Patients with anti-β1-AR Ab levels below the median (red) showed a lower event-free survival compared to patients with anti-β1-AR Ab levels ≥ median (blue; *p* = 0.049).

Five out of 11 patients with anti-β-AR Ab levels under the median reached the combined endpoint. Patients with antibody levels ≥ median experienced less adverse events, only 2 out of 11 patients (*p* = 0.361). The event-free survival using the combined endpoint of MCS, HTx, or death was significantly lower in MYC patients with anti-β-AR Ab levels below the median as compared to myocarditis patients with anti-β-AR Ab levels ≥ median (anti-β1/2/3-AR Ab: *p* = 0.049; Figure 4).

## Discussion

In this study, we investigated the anti-β-AR Ab levels in pediatric patients with biopsy-proven myocarditis according to different age groups and related outcomes.

We could detect anti-β-AR Ab in all pediatric myocarditis patients and controls, which underlines their pre-existence also in healthy or in patients without heart failure (19, 20). In the age group >5 years, anti-β3-AR Ab levels were significantly increased in patients with MYC as compared to CTRL. Throughout all age groups, anti-ß-AR Ab levels were consistently higher in patients with MYC as compared to CTRL, supporting the thesis of antibody increment in heart failure (21). However, the comparisons did not reach statistical significance in most age groups, this may be due to the small sample sizes. These results are in conflict with the ones of Simpson et al., where they did not find increased anti-β-AR Ab levels in children with myocarditis. An explanation could be that they did not group their cohort according to age, which might have been resulted in a non-significant difference in the whole cohort as compared to controls (12).

A possible mechanism in older myocarditis patients with higher anti-ß-AR Ab levels might be an innate Ab upregulation with an agonistic effect on the beta-adrenergic receptors in order to overcome cardiac dysfunction triggered by the myocardial inflammation. The initial upregulation might represent a normal immunological response rather than an autoimmunity process resulting in lower receptor expression and receptor desensitization and finally chronic heart failure (22–24). This could be supported by the fact that after ventricular assist implantation, anti-β1-AR Abs were undetectable in patients with DCM and increased anti-β1-AR Ab before implantation (25).

Our data cannot absolutely support the thesis of a protective effect of these antibodies described in other studies, but we found higher levels in the myocarditis age group of >5–17 years, which presented with less severe adverse events and better LVEF as compared to the other MYC age groups (19, 26). On the other end, the lowest antibody levels and worst disease courses with severely reduced left ventricular function were seen in patients <1 year of age. In this age group, the absence of a specific anti-β-AR Ab upregulation might be explained by inherently low overall IgG levels at this age. Therapeutic substitution of immunoglobulins might help to promote an immune response that resembles that of subjects with mature immune systems when applied early in this age group (2).

## Conclusion

There are age-dependent different anti-β-AR Ab levels in children with biopsy-proven myocarditis and controls. In myocarditis patients >5 years of age, anti-β3-AR Ab levels are significantly increased as compared to controls which might rather be compensatory to trigger a receptor agonistic effect than primarily upregulated as they do not present with severe heart failure. Especially children <5 years with lower anti-β-AR Ab levels experience more often adverse events, which might be a missing compensatory effect due to lower innate IgG levels and a potential therapeutic target for immunoglobulin substitution.

### Limitations

We analyzed, especially in the young age groups, a small number of patients and controls group. The size of these groups does not allow for drawing conclusions on the effects of anti-β-AR Ab on their outcome. Moreover, the children with myocarditis in the age group >5–17 years were older than the controls in this group. Additionally, even there was no myocardial inflammation or sign of heart failure, all controls had a simple isolated congenital heart defect, which could also have an influence on anti-β-AR Ab levels.

## Data Availability Statement

The raw data supporting the conclusions of this article will be made available by the authors, without undue reservation.

## Ethics Statement

The studies involving human participants were reviewed and approved by Institutional Ethics Committee Charité - Universitätsmedizin Berlin (EA2/131/10, EA2/074/13). Written informed consent to participate in this study was provided by the participants' legal guardian/next of kin.

## Author Contributions

FS, DM, and SS put down the concept and designed the study. FS analyzed the datasets, did the statistical analysis, wrote the initial draft, and finalized the article. CS helped design the study, analyze the datasets, and reviewed the manuscript draft. HH helped design the study, performed the measurements, analyze the dataset, and reviewed the manuscript draft. BO-R enrolled patients and reviewed the manuscript draft. TP was responsible for the ethics approval and biobanking. KK analyzed the biopsies. KK, FB, DM, and SS have reviewed, critically revised the initial, and final drafts of the manuscript. All authors contributed to the article and approved the submitted version.

## Funding

The planning and pilot phases of the MYKKE registry were funded through two project grants by Deutsche Herzstiftung e.V. (Germany). Since February 2017 MYKKE has been funded by kinderherzen – Fördergemeinschaft Deutsche Kinderherzzentren e.V. (Germany). In 2019 and 2020 there was financial support by the Berliner Sparkassenstiftung Medizin (2019-043) for this project. Logistic support and database management are provided by the Competence Network for Congenital Heart Defects (Germany), which received funding from the Federal Ministry of Education and Research, grant number 01GI0601 (until 2014), and the DZHK (German Centre for Cardiovascular Research) as of 2015.

## Conflict of Interest

HH is CEO and employed by CellTrend GmbH. The remaining authors declare that the research was conducted in the absence of any commercial or financial relationships that could be construed as a potential conflict of interest.

## Publisher's Note

All claims expressed in this article are solely those of the authors and do not necessarily represent those of their affiliated organizations, or those of the publisher, the editors and the reviewers. Any product that may be evaluated in this article, or claim that may be made by its manufacturer, is not guaranteed or endorsed by the publisher.

## Abbreviations

Anti-β-AR Ab: anti-beta-adrenergic receptor antibodies
ASD: atrial septal defect
CRP: C-reactive protein
CTRL: control group
DCM: dilated cardiomyopathy
DNA: Deoxyribonucleic acid
ELISA: enzyme-linked immunosorbent assay
EMB: endomyocardial biopsy
HTx: heart transplantation
IgG: immunoglobulin G
LVEF: left ventricular ejection fraction
LVIDd: left ventricular internal diastolic diameter
MCS: mechanical circulatory support
MYC: myocarditis group
NT-proBNP: N-terminal-pro brain natriuretic peptide
PDA: persistent ductus arteriosus
STEMI: ST-elevation myocardial infarct
RNA: Ribonucleic acid
(RT)-PCR: (real-time)-polymerase chain reaction.
